# An unveiling case of Nocardia pansinusitis in a patient with chronic lymphocytic leukemia: a case report

**DOI:** 10.1186/s13256-025-05037-0

**Published:** 2025-01-21

**Authors:** Maedeh Najafizadeh, Fatemeh Kourkinejad Gharaei, Reza Manouchehri Ardekani, Mahdi Rafiyan

**Affiliations:** 1 Infectious Diseases Research Center, Kashan University of Medical Sciences, Kashan, Islamic Republic of Iran; 2 Student Research Committee, Kashan University of Medical Sciences, Pezeshk Blvd. – Qotb Blvd, Kashan, Islamic Republic of Iran; 3 Autoimmune Disease Research Center, Kashan University of Medical Sciences, Kashan, Islamic Republic of Iran

**Keywords:** Sinusitis, Case report, Nocardia, CLL, Chemotherapy

## Abstract

**Background:**

*Nocardia* infections are rare infections in immunocompetent patients and occur mostly in immunocompromised individuals. Usually, *nocardia* affects skin, brain, and lungs, but in disseminated forms, which occurred mostly in immunocompromised patients, it can involve every organ. Nocardia sinusitis is extremely rare as our searches returned only a very few related studies.

**Case presentation:**

In this case report, we present, for the first time, a 55-year-old Iranian male patient diagnosed with chronic lymphocytic leukemia who was receiving chemotherapeutic drugs and developed a fever. Further laboratory tests and imaging revealed pansinusitis. Following rhinoendoscopy, the sinus mucosal biopsy pathology report showed sever inflammation accompanied by aggregation of filamentous thin-walled bacteria. The patient was treated with co-trimoxazole and meropenem. Following good clinical improvement the patient was discharged and advised to continue oral co-trimoxazole for 3 months.

**Conclusion:**

This case highlights that patients with febrile neutropenia should be assessed for rare infectious disease etiologies, especially those with chronic lymphocytic leukemia, as they have humeral immunodeficiency, and in the later stages of the disease, cellular immunodeficiency may also be involved. Therefore, a multisystem evaluation of patients with febrile neutropenia is necessary, particulary when no obvious source is identified in initial surveys, to uncover rare etiologies.

## Introduction

*Nocardia* species, with their Gram-positive, partially acid-fast bacilli, are widely distributed and found in various environments such as vegetables and soil as well as in both fresh and salt waters [5]. These filamentous, aerobic bacteria can cause localized or widespread infections, with the most commonly affected areas being the lungs, skin, and brain[14]. More than 50 species of *Nocardia* have been recognized, and six major taxa (including *N. nova complex, N. transvalensis complex, N. cyriacigeorgica, N. abscessus, N. farcinica,* and *N. brevicatena/N. paucivorans*) accommodate these species. *N. brasiliensis*, *N. nova*, and *N. farcinica* are the most common species isolated in the USA [5, 13, 22]. *Nocardia* usually affects the lungs, as its primary transmission route is through inhalation, but some studies have shown its dissemination even to the CNS through cutaneous inoculation [2, 3, 17]. It rarely causes infection in immunocompotent individuals and is considered as an opportunistic pathogen, having been seen in immunocompromised patients such as hematologic malignancies and organ transplant recipients. Its 1-year mortality is about 25% and can reach up to 50% in cases involving CNS [18]. Due to the non-specific clinical and radiological findings related to *Nocardia*, its diagnosis is challenging. In this study, we reported a case of *Nocardia* pansinusitis along with a review of the current literature on *Nocardia*-associated sinusitis.

### Case presentation

A 55-year-old Iranian male patient with a known case of CLL for 7 months was admitted to the hospital with fever and fatigue. He was a resident of Kashan city, Isfahan province, Iran, and was receiving the fludarabine, cyclophosphamide, rituximab (FCR) regimen (fludarabine (25 mg/m^2^; intravenous), cyclophosphamide(250 mg/m^2^; intravenous), and rituximab (375 mg/m^2^; intravenous)) for chemotherapy (six sessions) due to the more than 10% weight loss over 6 months, along with rapidly enlarging lymphadenopathy. His last full chemotherapy session was 3 months prior to this admission. He also received rituximab every 3 months (last dose, 17 days before admission, with the same dose as before). The patient’s fatigue started a week earlier and was accompanied by a non-productive cough, exertional dyspnea, and a headache in frontal region, along with a loss of appetite. The severity and frequency of the cough increased during this period. Fever began the day before admission. A toothache from 3 months ago was also noted in the patient’s history (the canine of right maxilla). Family history and psychosocial history were negative.

Upon examination, the patient presented with the following vital signs: a blood pressure of 92/78 mmHg, a pulse rate of 80 beats per minute, a respiratory rate of 18 breaths per minute, a temperature of 36.5 °C (axillary), and an oxygen saturation of 95% without supportive oxygen therapy.

Physical examination revealed posterior nasal drainage. There was no evidence of soft palate necrosis and petechiae. During chest auscultation, decreased breath sounds were detected on the left side of the chest, along with areas of increased tactile fremitus. Right ear otoscopy showed otitis media. Dental caries and tenderness in the gingiva of the canine tooth were detected, with no evidence of periodontitis.

Inflammatory biomarkers were the following: procalcitonin (PCT): 9, White Blood Cells (WBC): 710, neutrophils: 11.2%, Hemoglobin (Hb): 9.7, platelets: 134,000, Erythrocyte Sedimentation Rate (ESR): 105, and C-reactive protein (CRP): 242. As the patient’s absolute neutrophil count (ANC) was 79 and there was evidence of bacterial infection (high PCT level), broad spectrum intravenous antibiotics, including meropenem 1 g three times per day, vancomycin 1 g twice per day, and levofloxacin 750 mg daily, were initiated and his further evaluation was begun. All of the patient’s laboratory data are presented in Table 1.

**Table 1:** Laboratory finding at the time of admission and at the time of discharge

Lab test	Admission	Discharge	Reference range
WBC	710	3600	4000–7000
Absolute neutrophil count (ANC)	79	2592	–
Hb	9.7 g/dl	8.9 g/dl	13–16
PLT	134,000	72,000	150,000–450000
BUN	35 mg/dl	8 mg/dl	7–23
Cr	1.5 mg/dl	1.4 mg/dl	0.4–1.2
Aspartate aminotransferase (AST)	12 IU/L	–	37 <
Alanine transaminase (ALT)	40 IU/L	–	5–41
Alkaline phosphatase (ALP)	220 IU/L	–	in > 15 years 80–306
ESR	105 mm/h	60 ml/h	Up to 15
CRP	242 mg/dL	9 mg/dL	6
IgG	15 mg/dL	–	
(AFB smear & culture)*2		Negative	
MTB PCR		Negative	

**Fig. 1:**
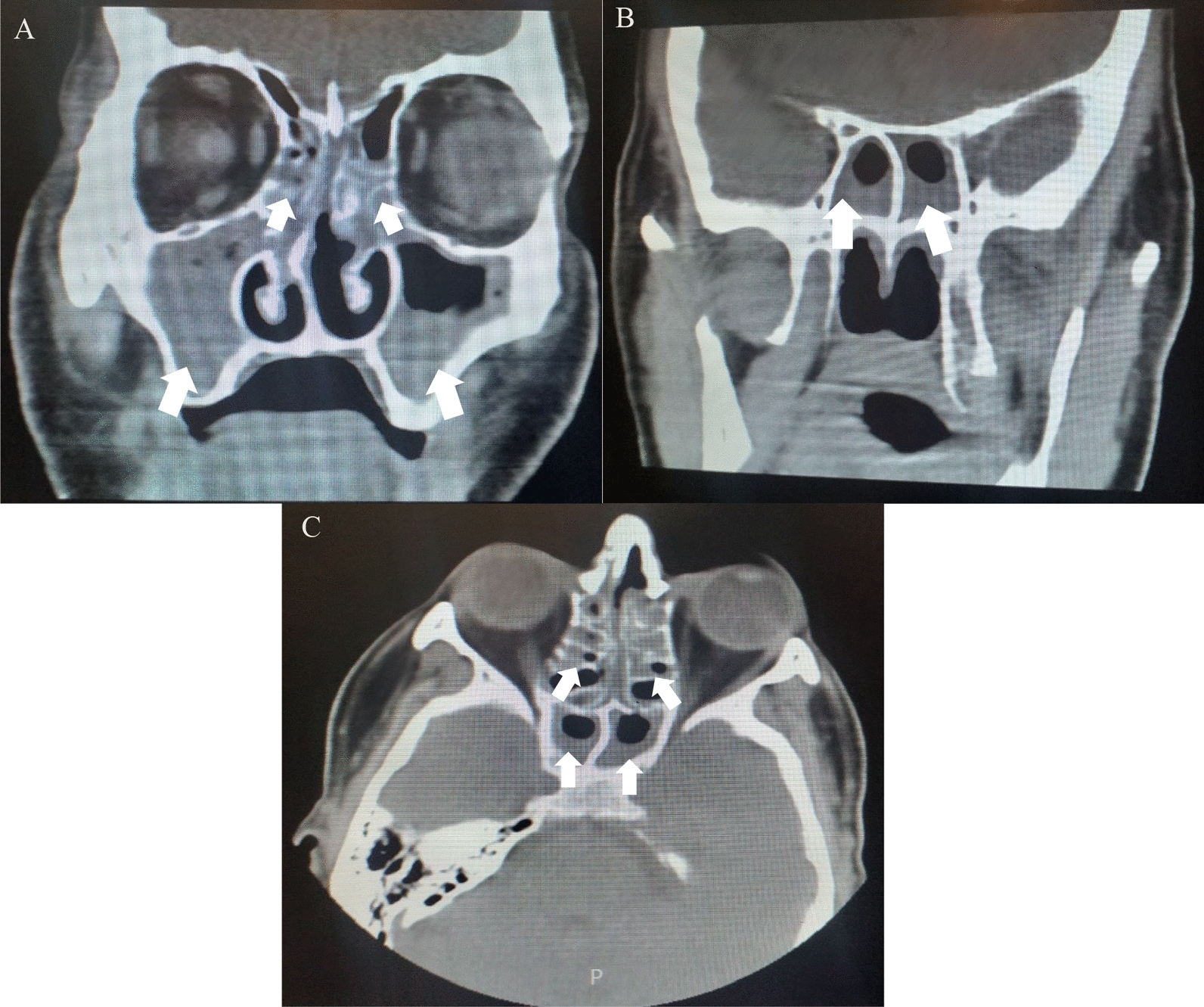
Paranasal sinuses computed tomography scan without contrast showing mucosal thickening of all paranasal sinuses. Note the fullness of all of the paranasal sinuses and their involvement as well as sinus edema (white arrows). **A**: coronal section of maxillary and ethmoid sinuses; **B**: coronal section of frontal sinuses; **C**: coronal section of paranasal sinuses showing ethmoid and sphenoid sinuses

In the workup, chest and paranasal sinuses computed tomography (PNS CT) scans were done 1 day after the patient’s admission. The chest CT scan did not reveal any pathological findings in either the heart or lungs. However, the PNS CT scan showed mucosal thickening of all paranasal sinuses. Additionally, septal deviation and bony spur formation were observed on the right side and the bilateral ostiomeatal complex (OMC) was closed.(Fig. 1). The patient was prepared for rhinoendoscopy. During the procedure (3 days after admission), no evidence of obvious tissue necrosis or fungal hyphae was seen by the HNENT specialist. The patient underwent bilateral antrostomy and middle concha resection the day after the rhinoendoscopy.

**Fig. 2:**
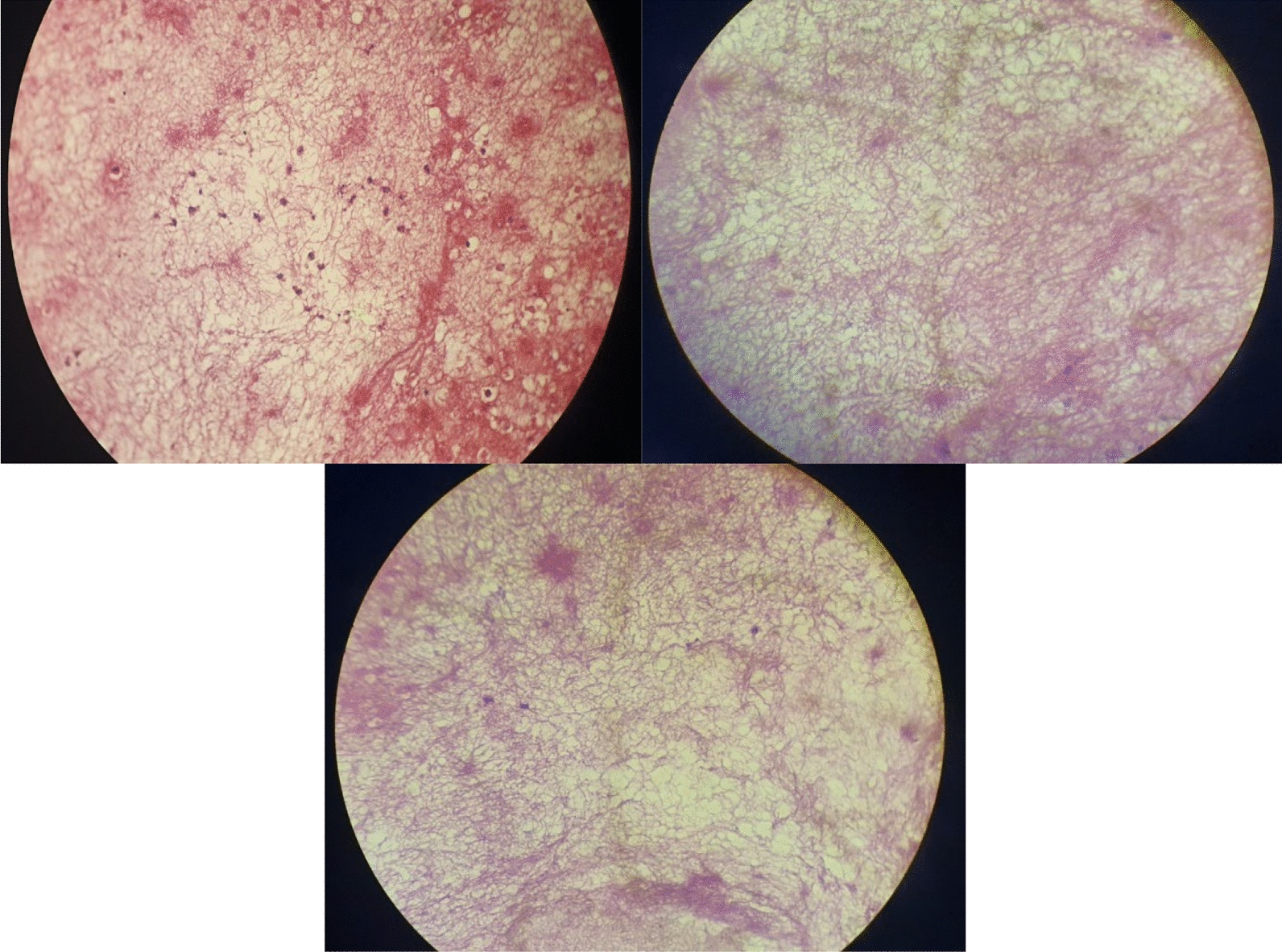
Microscopic view of sinus mucosal biopsy (hematoxylin and eosin stain). Note the darker spots showing inflammatory cells and bacterial aggregation in top left picture. The other two images also represent a network-like structure, which corresponds to the filamentous form of *Nocardia*

The pathology report of the nasal cavity and sinus mucosa biopsy was as follows: respiratory epithelium and mucosal glands. There was edema in the lamina propria with sever infiltration of acute and chronic phase inflammatory cells (lymphoplasmacytic cells, neutrophils, and eosinophils) as well as areas of bleeding and necrosis. In some sites, aggregation of filamentous Gram-positive, thin-walled bacteria was seen (compatible with *Nocardia* species). No fungal elements were identified in the specimen (Fig. 2).

With a total immunoglobulin G (IgG) of 15, the patient received intravenous immunoglobulin. On the basis of the pathology report, as described above, intravenous co-trimoxazole (7.5 mg/kg/daily) was initiated, and meropenem (1 g three times per day) continued. Vancomycin and levofloxacin were discontinued. A workup for brain involvement, including a brain CT scan, showed no signs of brain involvement. After the cessation of fever and improvement of neutropenia ( after 14 days from admission), the patient was discharged with good clinical status. Intravenous co-trimoxazole changed to oral type and was continued (160 mg/800 mg two times, daily) for 3 months. Follow-up after 3 months showed complete resolution of the signs and symptoms related to the *Nocardia* infection. Table 2 provides a comprehensive overview of the timeline of clinical events and the procedures that were carried out for the patient during his admission.

**Table 2:** Timeline of clinical events and procedures done for patient

Timepoint	Event description
T0—7 months	CLL diagnosed
T0—3 months	Last chemotherapy
T0—1 week	Fatigue + non-productive cough, exertional dyspnea and headache in frontal region along with loss of appetite started
T0—1 day	Fever started
T0	Initial presentation: 55-year-old male was admitted to the hospital with fever and fatigue, full physical examination, vital sign measurement, and laboratory tests were performed, and antibiotics initiated
T0 + 1 day	Chest and PNS CT scan were done
T0 + 3 days	Rhinoendoscopy performed
T0 + 4 days	Bilateral antrostomy and middle concha resection
T0 + 6 days	Pathology report of nasal cavity and sinus mucosa biopsy prepared, antibiotic regimen changed accordingly, and cotrimoxazole started
T0 + 7 days	Brain CT scan was carried out
T0 + 2 weeks	Fever cessation and symptom improvement
T0 + 3 months	Co-trimoxazole was discontinued

## Discussion

Despite the rareity of *Nocardia* cases, it can induce serious infectious disease associated with a high mortality rate. However, its nonspecific signs and symptoms, along with its potential to involve different parts of the body, which induce polymorphic clinical signs, make early diagnosis difficult. In this case, for the first time, we present a patient with pansinusitis due to *Nocardia* who was also receiving chemotherapy regimen for CLL. According to our search, there is no case report of *Nocardia* pansinusitis or a patient with CLL who developed *Nocardia* sinusitis, which indicates the importance of considering this diagnosis as a rare complication in immunocompromised patients such as patients with CLL.

Some clues could help physicians consider *Nocardia* sinusitis diagnosis: cases in immunocompromised patients, any disseminated or isolated nodular tissue lesions, suppurative granulomatous disease associated with community-acquired pneumonia, and age over 50 years could be suggestive of *Nocardia *[4, 6]. In this case, the patient was a 55-year-old man, immunocompromised due to both CLL and chemotherapy, who developed a productive cough, frontal headache, and fever.

As mentioned before, the main entry mode of *Nocardia* is through the respiratory system (spores/suspension of fragments of filaments), but it can also enter through mucous membranes and cutaneous wounds. After entry, it can induce septicemia and involved all organs. Precise laboratory tests and imaging with high sensitivity are important for excluding other opportunistic infections such as tuberculosis, *Cryptococcus, Histoplasma*, *Bacteroides, Aspergillosis*, and *Pneumococcus*, as well as ruling out concomitant brain abscess and lung infection [8]. In this case, it is possible that the main entry mode was his decayed tooth; his normal chest CT and the absence of lung involvement support this hypothesis.

*Nocardia* sinusitis is a very rare diagnosis in the literature, as a literature review returned only seven cases of *Nocardia* sinusitis summarized in Table 3 [4, 8, 11, 12, 15, 16, 19]. In most of these cases (five out of seven studies), a previous disorder such as diabetes, cancer, or human immunodeficiency virus (HIV) had weakened the immune system [4, 8, 12, 15, 19]. Similarly, in this case, the patient had CLL, which induces humeral immunodeficiency and further cellular immunodeficiency in later stages[9]. Thus, rare infectious diseases and other complications in immunocompromised patients [including patients who received chemotherapeutic agents, diabetes mellitus, patients with HIV/acquired immunodeficiency syndrome (AIDS), etc.] should always be considered by physicians. In these case reports, only a limited number of sinuses were involved, but in the present case report, the patient developed pansinusitis. Furthermore, he received fludarabine, which suppresses cellular immunity[7]. Additionally, cyclophosphamide, by inhibiting both cell-mediated and humoral immune responses, has extensive effects on the immune system [1]. Rituximab in this regimen suppresses humeral immunity and immune system activity [21]. Gram staining and imaging helped the authors reach the final diagnosis. On the basis of previous case reports, the core standard management and treatment of *nocardia* sinusitis is co-trimoxazole [4, 8, 12, 15, 16, 19]. Other antibiotics including imipenem, amikacin, erythromycin, or third-generation cephalosporins could be added to this regimen. However, antibiotic susceptibility testing is an important part of the treatment, as in recent years, *Nocardia* resistance to multiple antibiotics has been reported [10, 20]. As mentioned before, in parallel with other studies, we initiated cotrimoxazole and added carbapenem (meropenem) to this regimen, and after discharge, oral cotrimoxazole continued for 3 months.

**Table 3:** Case reports of *Nocardia* sinusitis

Gender/age	Past medical history	Clinical presentation/imaging	Other localization	Diagnosis	Species	Treatment/outcome	Reference
Female/72 years	Diabetes, breast cancer	Severe left frontotemporal headache and left trigeminal neuralgia along with increase of CRP (223)/CT scan indicated an abscess in the left pterygoid muscles and osteolysis of the greater wing of the left sphenoid bone with meningeal reaction and dissemination to the infratemporal fossa	Infratemporal fossa	*Nocardia nova* sphenoid sinusitis and infratemporal fossa abscess	*Nocardia nova*	Oral trimethoprim-sulfamethoxazole two tablets twice per day along with folic acid resulted in complete resolution of the infection, confirmed by neck, chest, and sinus CT at 6-month follow-up	[8]
Male/6 years	–	Headache, increased drowsiness, and double vision/ right orbital subperiosteal abscess and right maxillary and ethmoid sinusitis	Right orbital fossa	Bilateral maxillary and right ethmoid sinusitis	*Nocardia asteroides*	Trimethoprim-sulfamethoxazole 20 mg/kg/day orally every 6 hours, therapy for 7 months, and remained well 20 years after this disease	[16]
Male/43 years	Diabetes type 1	Nose block and a watery, non-foul smelling nasal discharge/HRCT of paranasal sinuses showed pansinusitis with left DNS with bony septal spur	-	*Nocardia nova* chronic maxillary sinusitis	*Nocardia nova*	Trimethoprim-sulfamethoxazole (TMP-SMX) for 6 weeks, which resulted in improvement of symptoms	[4]
Male/40 years	HIV	Chronic nasal obstruction/CT showed pansinusitis with a cyst in the left maxillary sinus	-	*Nocardia asteroids* sinusitis	*Nocardia asteroides*	Trimethoprim and sulfamethoxazole (TMP/SMX) for 2 months with an excellent outcome	[12]
Female/42 years	Recurrent episodes of maxillary sinusitis	Iridocyclitis, left maxillary sinusitis/CT scan showed mucosal hypertrophy of the left maxillary antrum, with an air-fluid level in this cavity	–	*Nocardia nova* maxillary sinusitis	*Nocardia nova*	A 6-week course of trimethoprim-sulfamethoxazole [two tablets (160/800 mg) every 12 hours] was begun and substituted with a 6-week course of erythromycin (every 6 hours)	[19]
Male/35 years	Renal transplant for reflux nephropathy	Headache, diplopia, increasing lacrimation, and nausea/soft tissue mass filling the sphenoid sinus and extending to the nasoethmoidal region on the left along with pituitary fossa and suprasellar cistern	Nasoethmoidal region on the left, pituitary fossa, and suprasellar cistern	*Nocardia asteroids* sphenoidal sinusitis	*Nocardia asteroides*	Oral sulfadimidine 1.5 g every 8 hours, amikacin (500 mg intravenously every 12 hours), co-trimoxazole (1600/320 mg intravenously every 6 hours), and imipenem (1.0 g intravenously every 8 hours) for a week, followed by 8 weeks of only imipenem; patient discharged with erythromycin changed to roxithromycin (300 mg twice daily) continued for 17 months and symptoms were improved	[15]
Female/39 years	–	Fever, rigors, diaphoresis, and night sweats/X-ray showed opacification of left maxillary sinus	–	*Nocardia asteroids* maxillary sinusitis	*Nocardia asteroides*	Oral sulfadazine, 1.5 g every 6 hours, and sodium bicarbonate, 650 mg four times daily for 5 months, resulted in improvement of symptoms	[11]

## Conclusion

Pansinusitis is a disease often associated with delayed diagnosis due to its polymorphic clinical features and insidious course. *Nocardia* infection of sinuses is extremely rare, and physicians must be familiar with the specific of management of this infection. This case showed that patients with febrile neutropenia should be assessed for rare infectious diseases etiologies, especially patients with CLL, who have both humeral and cellular immunodeficiency. Therefore, a multisystem assessment of patients with febrile neutropenia is necessary and should be considered in patients with no obvious source in primary surveys to find the rare etiology.

## Data Availability

Not applicable.

## Abbreviations

CLL: Chronic lymphocytic leukemia
PNS CT: Paranasal sinuses computed tomography
FCR: Fludarabine, cyclophosphamide, rituximab
AFB: Acid-fast bacillus
MTB: Mycobacterium tuberculosis
PCR: Polymerase chain reaction
PCT: Procalcitonin
OMC: Ostiomeatal complex
